# Synchronous Physical Telerehabilitation Versus Face-to-Face Rehabilitation in Axial Spondyloarthritis: A Focused Narrative Review

**DOI:** 10.3390/healthcare14203384

**Published:** 2026-10-11

**Authors:** Lorena Ciumarnean, Madalina Daiana Gavris, Stefan Cristian Vesa, Mircea Vasile Milaciu, Olga Hilda Orasan, Ioana Para, Teodora Gabriela Alexescu, Irina Maria Vlad, Gabriela Bombonica Dogaru

**Affiliations:** 1 Department of Internal Medicine, Iuliu Hatieganu University of Medicine and Pharmacy, 400012 Cluj-Napoca, Romania; lorena.ciumarnean@umfcluj.ro (L.C.); vasile.milaciu@umfcluj.ro (M.V.M.); hilda.orasan@umfcluj.ro (O.H.O.); ioana.para@umfcluj.ro (I.P.); teodora.alexescu@umfcluj.ro (T.G.A.); 2 Department of Hematology, The Oncology Institute Prof. Dr. Ion Chiricuta, 400015 Cluj-Napoca, Romania; 3 Department of Pharmacology, Iuliu Hatieganu University of Medicine and Pharmacy, 400012 Cluj-Napoca, Romania; stefanvesa@gmail.com; 4 Department of Neurology, Iuliu Hatieganu University of Medicine and Pharmacy, 400012 Cluj-Napoca, Romania; vlad.irina.maria@elearn.umfcluj.ro; 5 Department of Medical Rehabilitation, Iuliu Hatieganu University of Medicine and Pharmacy, 400012 Cluj-Napoca, Romania; dogarugabrielaumf@gmail.com

**Keywords:** ankylosing spondylitis, axial spondyloarthritis, exercise therapy, face-to-face rehabilitation, physiotherapy, synchronous telerehabilitation

## Abstract

**Background and Objectives**: Exercise is central to axSpA management, but whether live remote delivery preserves effects of the same face-to-face program is uncertain. **Methods**: Five databases were searched until 9 September 2026; registry, reference, and citation searches ended on 11 September 2026. Reports from one trial were linked; contextual studies were illustrative. **Results**: Of 214 database records, 123 unique records were screened, and 11 reports representing eight studies were selected from the five databases. A ClinicalTrials.gov record and doctoral thesis linked to the Karaca trial were obtained separately and were not counted among those 11 database-selected reports. One assessor-blinded RCT compared the same individualized clinical Pilates program by videoconference or in clinic. Fifty participants were randomized, 46 assessed at eight weeks, and 40 at six months. Median BASFI improvement was 3.0 versus 2.5 points at eight weeks (*p* = 0.587) and 2.0 versus 2.0 points at six months (*p* = 0.838). Mann–Whitney U tests used α = 0.017; between-group effect estimates and 95% confidence intervals were unavailable. All-randomized attendance was 93.8% versus 94.0%. Five participants reported transient symptoms; three serious events were judged unrelated, and no intervention-related serious adverse event was reported. **Conclusions**: Remote delivery was implementable with high short-term attendance among digitally eligible participants. This trial does not establish equivalence, non-inferiority, preserved effect, broad accessibility, or a safety profile. Adequately powered non-inferiority trials are needed.

## 1. Introduction

Axial spondyloarthritis (axSpA) is a chronic inflammatory rheumatic disease involving the sacroiliac joints and spine, with radiographic and non-radiographic forms. Pain, stiffness, fatigue, reduced mobility, and impaired function can affect quality of life and work participation [1]. Current international recommendations combine pharmacological treatment with education, individualized physiotherapy, and regular exercise [2].

Rehabilitation is therefore not an optional adjunct but a core component of disease management. Randomized trials and evidence syntheses report improvements in disease activity, pain, mobility, physical function, fatigue, and health-related quality of life, although effects differ according to intervention content, intensity, supervision, and outcome [3,4].

Face-to-face rehabilitation can be limited by travel, work, cost, disability, or geography. Telerehabilitation may reduce some of these barriers, but access to a suitable device, a stable internet connection, sufficient digital literacy, and a safe home environment can create others [5,6]. Synchronous telerehabilitation enables real-time observation, feedback, and exercise correction, whereas asynchronous programs use recordings, apps, or written material without immediate interaction. Because these formats differ in supervision and adherence support, they should not be treated as a uniform intervention [7,8]. Safety and implementation also depend on patient selection, clinical judgment, technical reliability, and systematic adverse-event monitoring [6,9,10].

A recent narrative review considered telerehabilitation in ankylosing spondylitis broadly [11], but a clinical substitution question requires a narrower comparison: the same supervised rehabilitation intervention delivered synchronously at a distance versus face-to-face. Trials comparing remote exercise with usual care, a wait list, prerecorded exercise, or a different treatment address related but different questions.

Accordingly, this focused narrative review aimed to (1) summarize reports identified within the focused search that directly compared synchronous telerehabilitation with supervised face-to-face rehabilitation; (2) distinguish direct, indirect, mixed-mode, and illustrative contextual evidence; (3) appraise the internal validity and interpretive limits of the reported comparisons; and (4) identify implications for future comparative research.

## 2. Materials and Methods

### 2.1. Focused Literature Search

Searches were designed around three concepts: axSpA/ankylosing spondylitis, remote delivery, and physical rehabilitation or exercise. The initial searches were executed by Gabriela Parjol, using a strategy developed by M.D.G., in PubMed/MEDLINE, Cochrane CENTRAL, PEDro, Scopus, and Web of Science from inception to 29 April 2026. The same five databases were rerun from inception on 9 September 2026 without date, language, or study-design restrictions, using the database-specific strategies reproduced in Supplementary Section S1. The update retrieved 214 records: 30 from PubMed, 10 from CENTRAL, four from PEDro, 98 from Scopus, and 72 from Web of Science. Identifier-prioritized deduplication using DOI, PMID, and normalized titles, followed by comparison of non-identical title variants, removed 91 duplicate copies and left 123 unique bibliographic records. Machine assistance was limited to deduplication, reconciliation of bibliographic identifiers and title variants, record organization, and generation of the audit trail; no record was automatically included or excluded. All final inclusion/exclusion decisions and reason codes were determined by human reviewers. One hundred eleven records were excluded at title/abstract screening; 12 reports were assessed in full or expanded form, all were retrieved, and one was excluded. Thus, 11 reports representing eight studies were selected from the five databases. The complete record-level audit is supplied as Supplementary Data S1, and the full-text exclusion is reported in Supplementary Section S2. Initial study-selection decisions were made by one review author in Rayyan (Rayyan Systems Inc., Cambridge, MA, USA). During revision, a second review author (M.D.G.) independently verified the eligibility decisions and exclusion coding for all 123 unique records against the prespecified evidence categories. No discrepancies were identified; therefore, no consensus adjudication was required.

ClinicalTrials.gov, reference-list, linked-report, and forward-citation checks were updated on 11 September 2026. The two-day difference from the database cut-off reflects the sequential processing of the database exports; these supplementary searches could add, but could not remove, eligible reports. The exact ClinicalTrials.gov query returned 10 registry records. The ClinicalTrials.gov record and doctoral thesis linked to the Karaca trial were obtained separately through the registry and linked-report searches and were not counted among those 11 database-selected reports. Forward-citation searching in OpenAlex (OurResearch, Sanford, NC, USA) across nine seed reports returned 50 unique citing works. No additional completed trial directly comparing matched synchronous and face-to-face rehabilitation was retained. One ongoing contextual registry (NCT07503327) compared live WhatsApp exercise, prerecorded exercise, and a brochure, but had no face-to-face arm and no results. Reference lists of the retained outcome reports were also checked. Search strings, dates, yields, procedures, and selection counts are provided in Supplementary Sections S1 and S2.

### 2.2. Scope and Categories of Evidence

Direct comparative evidence had to include adults with axSpA or ankylosing spondylitis, a physical rehabilitation intervention delivered live via videoconference, and a supervised face-to-face comparator. Randomized and non-randomized studies, conference abstracts, registry entries, and these were accepted because of the small volume of direct evidence. Where reports on populations with other conditions provided axSpA-specific data, those data were included. Where a particular trial yielded multiple associated reports or publications, all documents were linked and treated as one study rather than as separate studies; associated registries, protocols, and other documentation were treated as reports linked to that study rather than as independent efficacy studies.

Contextual reports were used illustratively rather than comprehensively. From reports encountered in the focused database, reference, citation, and reviewer-suggested searches, an example was eligible when it (1) reported adult axSpA- or ankylosing-spondylitis-specific results; (2) included a live remote exercise or rehabilitation component, alone or within a mixed program; (3) had a comparator that informed feasibility, attendance, adverse-event reporting, or the contribution of real-time supervision; and (4) reported outcome data rather than only a protocol or registry entry. Studies were not retained as a complete sample of every report satisfying these features. Consequently, the contextual set cannot support prevalence estimates, comparative safety conclusions, or a claim that it is balanced or exhaustive. Purely asynchronous and non-comparative reports were cited only when directly relevant to implementation or maintenance.

Once the collected reports had been compiled, they were grouped thematically according to their relevance to the research question, mode of delivery, and comparator for analysis using narrative synthesis. Direct reports compared outcomes in trials specifically designed to assess synchronous versus face-to-face delivery. Indirect reports involved a modality comparison that was not randomized. Mixed-mode reports involved either blended synchronous–asynchronous methods or differences in both intervention content and delivery modality. Contextual research did not support inferences about substitution for face-to-face rehabilitation.

No report was excluded solely because of language. At title/abstract screening, all 123 unique database records were assessed using the titles, abstracts, and indexing metadata supplied by the databases, including English-language titles or abstracts where available for non-English publications. Records that were clearly ineligible from this information were excluded for the substantive reasons recorded in Supplementary Data S1, not because of language. When the supplied information was insufficient to exclude a record confidently, the report was advanced for fuller assessment irrespective of language. The Karaca doctoral thesis was the only potentially eligible non-English source for which full-text translation was required. During revision, the full thesis was retrieved from the official Hacettepe University repository. The extraction in Supplementary Section S4 was prepared by machine-assisted translation of the Turkish text and direct transcription from the original tables. The source page, table, denominator, analysis population, time point, method, and estimate are recorded for each principal item. Before resubmission, an independent authorized translator proficient in Turkish and English compared the methodological descriptions, outcome labels, and all transcribed numerical data with the Turkish original on the pages specified in Supplementary Section S4. The translator confirmed the accuracy of the translations and extracted data; any wording corrections identified during verification were incorporated.

### 2.3. Data Extraction, Synthesis, and Critical Appraisal

L.C. and M.D.G. initially extracted study design, participant flow, intervention dose and delivery, comparator, follow-up, outcomes, attendance, adverse events, and limitations collaboratively rather than as independent duplicate extractions; M.D.G. checked numerical values against the reports then available. No separate extraction file was retained during the initial review. During revision, a structured source-level extraction table was created for the Karaca thesis (Supplementary Section S4). Between-group results were preferred to within-group changes. Confidence intervals were transcribed where reported and were not reconstructed from medians, interquartile ranges, or *p* values when the required assumptions or data were unavailable. Reports from the same study were reconciled rather than counted as independent evidence. Meta-analysis was not attempted because only one directly comparative RCT was identified within the focused search and the remaining studies differed materially in design, comparator, content, and delivery format.

RoB 2 was applied to the between-group BASFI result at the end of the intervention in each randomized study, thereby using a common outcome rule across trials; the six-month BASFI result from the direct Karaca trial was assessed separately because durability was central to the review question. The estimand of interest was the effect of assignment to intervention [12]. The original 2016 ROBINS-I tool was applied to the non-randomized delivery-mode contrasts in Fayet and Bundle [13]. The principal endpoint was chosen because it coincided with completion of the assigned program; the additional Karaca endpoint assessed maintenance. The Karaca registry version history, abstracts, and thesis were considered together for selection of the reported result. One review author completed all assessments during revision. The assessments were not independently duplicated, and disagreement resolution was therefore not applicable. Detailed source-based answers and domain rationales are supplied in Supplementary Section S6.

## 3. Results

The Results first map the identified evidence, then reconstruct the direct Karaca trial across its linked reports, examine the result-level risk of bias, and finally describe indirect, mixed-mode, and illustrative contextual evidence.

### 3.1. Evidence Map

The database update retrieved 214 records; 91 duplicate copies were removed, 123 unique records were screened, 12 reports were assessed in full or expanded form, and 11 reports representing eight studies were selected from the databases. The ClinicalTrials.gov record and doctoral thesis linked to the Karaca trial were obtained separately through supplementary searches and were not counted among those 11 reports. No second randomized trial designed to compare the same supervised intervention delivered synchronously by videoconference or face-to-face was identified within this focused search. The direct evidence comprised one randomized clinical trial of the same supervised clinical Pilates intervention. That trial generated a preliminary abstract, an interim abstract, a registry record with four historical versions, and a doctoral thesis [14,15,16,17]. An exploratory non-randomized comparison of face-to-face and remote Tai Chi delivery was also identified [18].

A 2026 yoga trial compared live group instruction with individual physiotherapy, but the online intervention involved a mix of live and prerecorded content, while the groups also differed in intervention content and dose [19].

Five randomized trials with other comparators were retained as illustrative contextual examples [20,21,22,23,24]. They were not selected through a protocolized comprehensive sampling process and should not be read as a complete evidence base. Table 1 maps the different questions these studies address.

### 3.2. Direct Comparative Evidence: The Karaca Trial

The reporting chronology changes the amount, but not the identity, of the direct evidence. The 2024 preliminary abstract reported 20 participants [14]. The 2025 interim abstract stated that 46 participants had entered and presented post-intervention data for 38 and follow-up data for 32 [15]. The completed registry record reported 50 participants [16]. The doctoral thesis, deposited in the Hacettepe University repository in October 2025, reported 50 randomized participants, 46 post-intervention assessments, and 40 six-month assessments [17]. These are sequential reports from one trial, not independent efficacy studies.

The completed study was a single-center, assessor-blinded, parallel-group RCT in which both groups received the same individualized clinical Pilates program. The only intended difference was live videoconference delivery at home versus in-clinic supervision. Table 2 provides further details about the population and intervention needed to assess applicability.

The thesis designated BASFI as its primary outcome. In the ITT dataset, median BASFI values in the face-to-face group at baseline, post-treatment, and follow-up were 4.2 (IQR 2.8–5.7), 1.0 (0.5–2.0), and 2.0 (0.7–2.7), respectively. Corresponding values in the telerehabilitation group were 3.3 (2.4–5.2), 0.7 (0.0–1.5), and 1.0 (0.3–2.0). These within-group trajectories do not by themselves estimate the comparative effect of delivery mode.

Median improvement from baseline was 3.0 (IQR 1.1–4.3) versus 2.5 (1.8–3.4) points after treatment (*p* = 0.587), and 2.0 (1.0–3.7) versus 2.0 (1.1–3.2) points at follow-up (*p* = 0.838), face-to-face versus telerehabilitation. The thesis compared participant-level change scores between groups with Mann–Whitney U tests using a Bonferroni-adjusted significance threshold of α = 0.017 for the three pairwise time contrasts. The reported *p* values test a distributional/rank contrast in change scores; they are not estimates of a difference between group medians. Numerical between-group effect estimates and 95% confidence intervals were not reported and could not be reconstructed reliably from the published medians, IQRs, and *p* values alone [17].

Similarly, for the central secondary outcomes, mean BASDAI values at baseline, post-treatment, and follow-up were 5.52 ± 2.04, 2.32 ± 1.45, and 3.52 ± 2.46 in the face-to-face group and 4.59 ± 1.85, 1.77 ± 1.06, and 2.13 ± 1.05 in the telerehabilitation group; the group-by-time interaction was not statistically significant (*p* = 0.313). Median BASMI improvement was 0.8 versus 0.8 points both post-treatment (*p* = 0.876) and at follow-up (*p* = 0.969). Median ASQoL improvement was 4.0 versus 4.0 points post-treatment (*p* = 0.689) and 4.0 versus 4.0 points at follow-up (*p* = 0.946). Exact 95% confidence intervals for these between-group comparisons were not tabulated [17].

Attendance was 93.8% in the face-to-face group and 94.0% in the telerehabilitation group when all randomized participants were included; among intervention completers, corresponding values were 98.2% and 98.4%. These descriptive rates apply to a digitally selected population: eligibility required the participant or a helper to be able to use a smartphone or computer and required a smartphone or computer with an active internet connection. The flow diagram reported 62 individuals who did not meet eligibility criteria and 77 who declined, but did not separate technology-, internet-, home-environment-, or delivery-preference-related reasons. Five participants reported transient increases in pain or muscle cramps (three face-to-face and two telerehabilitation) without interruption of subsequent sessions. Two serious events during treatment and one during follow-up were judged unrelated to the intervention; no intervention-related serious adverse event was reported. The sample size and eight-week exposure are insufficient to establish a safety profile or exclude uncommon harm [17].

Both groups showed numerical improvement, and the small superiority-designed trial did not detect a statistically significant between-group difference. These observations do not establish equivalence or non-inferiority. The sample-size calculation targeted a repeated-measures group-by-time interaction; no non-inferiority margin was prespecified, and no confidence interval around the delivery-mode contrast was reported. A clinically important disadvantage of either mode therefore cannot be excluded. 

Changes in the trial registration over time and their implications for interpreting the study findings are summarized in Table 3.

### 3.3. Risk of Bias in the Comparative Results

The completed single-assessor judgments are summarized in Table 4, with the source-based signaling answers and domain rationales in Supplementary Section S6. RoB2 yielded some concerns for the Karaca BASFI result at both eight weeks and six months. Sequence generation and allocation concealment were adequate, but missing outcome data, an unblinded self-reported outcome, incomplete reporting of the multiple-imputation model, and uncertainty arising from registry changes and the absence of a dated public analysis plan reduced confidence. The registry initially specified three intervention groups and an estimated sample of 30; on 17 July 2024, after recruitment had begun, it was changed to two groups and an estimated sample of 50. BASDAI and BASFI were both primary outcomes from the first registry version, although their order was reversed in 2024 and the thesis later called BASFI the sole primary outcome. These facts indicate uncertainty, not proof, regarding selection of the reported result. Among the contextual randomized studies, Acar was judged as having some concerns; Singh, Gencer, Sen, and Yentur were judged at high risk, principally because of post-randomization exclusions or missing outcomes related to adherence, incomplete allocation/reporting information across several domains, or both. The Fayet delivery-mode contrast was at critical risk in ROBINS-I because delivery mode was inseparable from calendar period and the COVID-19 context; Bundle was at serious risk because allocation depended on preference or existing care and the intervention packages differed in delivery, content, and dose.

### 3.4. Indirect and Mixed-Mode Comparative Evidence

Forty adults with stable axSpA were recruited by Fayet et al. into Tai Chi programs delivered in hospital (n = 20) or remotely at home (n = 20) [18]. They were randomized to immediate Tai Chi or a waiting-list period, not to remote or in-hospital delivery. In-hospital sessions were delivered from January 2019 to March 2020, whereas a separate at-home program was implemented in 2021 during the COVID-19 pandemic and matched to the earlier in-hospital cohort by age and sex. The exploratory modality comparison found no statistically significant difference in total physical activity at month 2. However, it is impossible to separate the effect of delivery mode from that of calendar period and pandemic conditions. Moreover, baseline characteristics were presented for the immediate-treatment and waiting-list groups rather than for the remote and in-hospital cohorts. Attrition in the in-hospital and at-home cohorts is not fully reconciled between the participant-flow diagram and the per-protocol tables. The comparison should therefore be interpreted as indirect evidence.

Bundle et al. compared an eight-week online yoga program in 24 participants with axSpA with routine individual physiotherapy in another 24 participants; 28 healthy adults formed a separate reference group [19]. Allocation depended on participant preference or existing physiotherapy. Yoga could be attended live or via recordings, whereas physiotherapy was not standardized and had a lower minimum weekly duration. Numerical changes, including BASMI −0.31 versus +0.29 and BASFI −0.17 versus +0.05, cannot isolate delivery mode from content, dose, self-selection, or synchro-nous/asynchronous access.

### 3.5. Contextual Randomized Evidence

Three trials evaluated remote exercise compared with a wait-list, standard care, or home exercise. Acar et al. randomly allocated 60 adults with ankylosing spondylitis to attend three weekly live, small-group online yoga sessions via Zoom for eight weeks or to a wait-list; 55 participants were analyzed. Mean BASFI changes were −1.321 (95% CI −1.782 to −0.861) and −0.178 (−0.647 to 0.291) in the tele-yoga and control groups, respectively (group-by-time interaction, *p* = 0.001). The intervention group attended a mean of 22.07 of 24 sessions, and no yoga-related adverse events were reported [20].

Singh et al. analyzed 109 participants after a three-month program delivered in a mixed-modality format that included two synchronous, remotely guided yoga classes per week, as well as recordings and image materials. Between-group change estimates for tele-yoga versus control were −0.79 (95% CI −1.30 to −0.29) for BASDAI and −0.86 (−1.59 to −0.14) for BASFI. Because of the mixed delivery format, the results cannot be attributed specifically to the synchronous component of the remotely delivered program [21].

Gencer et al. randomly allocated 68 participants and analyzed 59 after Baduanjin qigong delivered by telehealth or home exercise for 12 weeks. Between-group change-score tests were reported for BASFI (*p* = 0.008), sleep quality (*p* = 0.018), and lumbar flexion (*p* = 0.046), without confidence intervals [22].

Two trials examined synchronous compared with asynchronous delivery. Sen et al. randomly allocated 50 adults and analyzed 44. BASFI improved by 1.68 points in the live remote group and by 0.48 points in the asynchronous group (*p* = 0.025), while adherence was reported to be higher with the live program: 95% versus 70% [23].

Yentur et al. randomized 44 adults and analyzed 39. Post-program BASFI was 3.60 in the live group and 3.40 in the YouTube group (*p* = 0.799); most other between-group comparisons were not statistically significant. The flow diagram lists four withdrawals, but five randomized participants were omitted from the analysis, leaving one participant unexplained [24]. These trials indicate that real-time supervision may contribute to maintaining program engagement or improving selected outcomes compared with unattended prerecorded content, although they cannot establish comparative efficacy versus live face-to-face rehabilitation.

## 4. Discussion

The Discussion separates what the direct comparison can establish from what indirect and illustrative evidence can only suggest, before considering applicability and future research priorities.

### 4.1. Principal Interpretation

Within the focused search, one small single-center RCT directly compared synchronous and face-to-face delivery of a matched intervention. It documents improvement within both groups and high attendance among participants who met digital eligibility criteria, while no intervention-related serious adverse event was reported. It does not demonstrate that remote delivery preserves the face-to-face effect, is equivalent or non-inferior, is accessible to the wider axSpA population, or has an established safety profile. Indirect and illustrative contextual studies answer different questions and do not close this evidence gap.

Statistical non-significance means that “no statistically significant difference was found,” not that “the treatments are the same, comparable, or equivalent.” Non-inferiority requires adequate power, a clinically and statistically justified margin, confidence intervals interpreted against that margin, and consistent intention-to-treat and per-protocol analyses [25,26]. The Karaca thesis used an interaction-based sample-size calculation and conducted multiple tests with some adjustment for multiple comparisons, but did not use the design features described above [17]. These findings are encouraging regarding feasibility, but cannot justify the routine substitution of synchronous telerehabilitation for face-to-face rehabilitation.

### 4.2. Why Evidence Categories Matter

The Tai Chi and yoga trials demonstrate why an evidence hierarchy is crucial. In Fayet et al., delivery mode cannot be separated from calendar period and the context of the COVID-19 pandemic [18]. In Bundle et al., intervention content, group preference, weekly dose, and even the type of supervision—synchronous versus asynchronous and live versus prerecorded—were confounded [19]. Both studies can demonstrate that remote mind–body exercise is feasible, but neither can isolate the contribution of delivery mode. Trials comparing an intervention with a waiting list or standard care estimate the additional value of the exercise program, whereas synchronous-versus-asynchronous trials estimate the value of real-time supervision. These are useful but different questions.

However, these trials also show that “telerehabilitation” is not a single intervention. There are many distinct types of physical training that can be delivered remotely: yoga, Pilates, Tai Chi, Baduanjin, multicomponent exercise, core-stability training, and others. Each has different physiological targets and requirements for progression, frequency, equipment, and technique. Depending on the type of supervision and delivery mode, remote exercise and support can consist of one-to-one live coaching, remotely supervised group training, prerecorded online sessions, or combinations of these formats. Future comparisons should distinguish exercise content, supervision, dose, group size, feedback, home equipment, and asynchronous co-interventions.

### 4.3. Feasibility, Safety, and Clinical Applicability

The Karaca intervention and the live arms of Acar and Sen had high short-term attendance, indicating high engagement among enrolled participants and supporting the practical implementability of these programs [17,20,23]. Attendance alone does not establish acceptability, because direct information on satisfaction, delivery preferences, willingness to continue, and reasons for withdrawal or non-participation was limited or unavailable. In Karaca, 77 individuals declined participation, but the available flow information does not distinguish technology-, home-environment-, or delivery-preference-related reasons. Enrollment required a smartphone or computer with an active internet connection and the participant’s or a helper’s ability to use the technology. High attendance after this digital selection should not be interpreted as evidence of population-wide accessibility. Moreover, eight- to twelve-week programs do not establish long-term adherence. In a subsequent 12-month cohort of 50 people with axSpA, mean adherence declined from 44% in the first four-week period to 19% in the final four-week period, and only 12 participants met the prespecified long-term adherence threshold [27].

These findings highlight the need for longer-term follow-up and active adherence-support strategies and suggest that future trials should include follow-up extending to 6–12 months [27].

Future reports should describe device and connectivity requirements, assistance needed, technical failures, and the characteristics of otherwise eligible people who could not or did not wish to participate. The Karaca thesis usefully separated transient symptoms from serious events judged unrelated to the intervention, but its small sample and short treatment period cannot establish safety. Across the wider literature, adverse-event ascertainment and reporting varied, and absence of a reported event is not evidence that none occurred [9]. Physical therapy guidance emphasizes patient selection, privacy, emergency preparedness, and clinical judgment [6]. Future axSpA trials should pre-specify adverse-event definitions and systematically monitor symptom exacerbations, falls, and technical incidents.

The evidence is insufficient to recommend routine substitution of synchronous telerehabilitation for face-to-face rehabilitation. The direct trial shows only that an individualized program could be delivered remotely to selected, ambulatory, digitally eligible participants with high attendance over eight weeks. This supports further comparative evaluation and shared consideration of delivery preferences; it is not evidence of equivalent clinical effectiveness or broad scalability. Hands-on examination or treatment, diagnostic uncertainty, inadequate video quality, or an unsafe home environment may still require in-person care [6,10].

Hybrid care likewise requires direct evaluation rather than assuming that evidence for either component transfers to the combination. Any clinical implementation should distinguish a feasibility decision for an individual patient from an evidence-based claim that remote delivery can replace matched face-to-face rehabilitation.

### 4.4. Future Research Priorities

A definitive substitution question requires an adequately powered, preferably multi-center non-inferiority RCT in which exercise content, therapist contact time, dose, progression rules, and co-interventions are matched and delivery mode is the intended difference. The non-inferiority margin must be prespecified and justified clinically and statistically, with confidence intervals interpreted against that margin [25,26]. Both ITT and PP analyses should be prespecified and conclusions should be consistent across them.

The trial should also use concealed allocation, blinded outcome assessment where feasible, a prespecified missing-data strategy, and multiplicity control. The primary outcome and assessment time point should be justified in advance. Longer follow-up should measure maintenance, while implementation outcomes should include attendance, technical failures, adverse events, satisfaction, access barriers, costs, and healthcare use. Table 5 consolidates these priorities.

### 4.5. Limitations of This Review

This review used a focused narrative approach. The initial search was targeted and initial study selection was conducted by one reviewer. During revision, the same five databases were rerun to 9 September 2026, yielding 214 records and 123 unique records after deduplication. Machine assistance was limited to deduplication, record organization, reconciliation of bibliographic identifiers and title variants, and generation of the record-level screening log and full-text exclusion record; it did not make final eligibility decisions. No record was automatically included or excluded, and all final eligibility decisions and reason codes were determined by human reviewers. Registry, reference, and citation checks were completed on 11 September 2026. A second review author independently verified the eligibility decisions and exclusion coding for every one of the 123 records, and no discrepancies were identified. Because this verification was retrospective rather than prospective parallel dual screening, it remains less robust than fully independent screening performed concurrently by two reviewers. In addition, the Web of Science export did not preserve the individual citation indexes selected, PEDro provided an aggregate four-record workbook rather than separate raw yields for each of four simple queries, and cited-reference counts were not recorded. The evidence-scarcity conclusion therefore remains explicitly limited to this focused search. The illustrative contextual set was not comprehensive or protocolized and cannot represent the full balance of remote-exercise evidence in axSpA.

The most complete report of the direct trial is a doctoral thesis written predominantly in Turkish. A structured machine-assisted working translation and extraction was prepared during revision from the official full text and was independently checked against the Turkish original by an authorized translator proficient in Turkish and English. The verification covered the methodological descriptions, outcome labels, groups, denominators, analysis populations, time points, statistical tests, and numerical values listed in Supplementary Section S4. The translator confirmed the accuracy of the translated and transcribed information, and any wording corrections were incorporated. The thesis has not undergone the peer review associated with a full journal article.

The submitted version did not retain completed risk-of-bias forms. During revision, one review author completed the RoB 2 and ROBINS-I assessments using the prespecified result-selection rule and source-based domain rationales in Supplementary Section S6. The assessments were not independently duplicated, so disagreement resolution did not apply; this single-assessor procedure may increase subjectivity and is a methodological limitation. No formal certainty-of-evidence assessment was undertaken. Meta-analysis was inappropriate because only one direct randomized comparison was identified within the focused search and the remaining studies were heterogeneous.

## 5. Conclusions

Within this focused search, one small RCT directly compared a matched synchronous telerehabilitation program with face-to-face delivery. It documents that the remote program was implementable and achieved high attendance over eight weeks among digitally eligible participants. Both groups showed numerical improvements, and no statistically significant between-group difference was detected, but the study does not establish that remote delivery preserves the face-to-face effect. Five participants experienced transient pain or muscle cramps, three serious events were judged unrelated, and no intervention-related serious adverse event was reported; these observations do not establish a safety profile.

Equivalence and non-inferiority remain unproven. The study used a superiority framework, did not prespecify a non-inferiority margin, and did not report a confidence interval for the principal delivery-mode contrast. Its high attendance cannot be generalized to people without the required device, connectivity, digital capability or assistance, or a suitable home environment.

Indirect, mixed-mode, and illustrative contextual studies support the practical possibility of delivering exercise remotely but do not determine whether synchronous telerehabilitation can replace face-to-face rehabilitation. Routine substitution is not supported. Multicenter non-inferiority trials are required, with matched intervention parameters and clinically interpretable estimates of effectiveness, durability, adverse events, access, patient experience, and costs.

## Figures and Tables

**Table 1:** Evidence map for physical telerehabilitation in axial spondyloarthritis.

Evidence Category and Source	Design, Sample, and Comparator	Main Finding	Interpretive Value
Direct: Karaca et al. [14,15,16,17]	Assessor-blinded RCT; 50 randomized to the same individualized program for 60 min, 3 times/week for 8 weeks; 6-month follow-up.	BASFI improvement: 3.0 versus 2.5 points after treatment (*p* = 0.587) and 2.0 versus 2.0 at follow-up (*p* = 0.838); between-group effect estimates and 95% CIs were not reported.	Only trial designed for the main question identified within the focused search. Final results are in a doctoral thesis; no non-inferiority margin was prespecified.
Indirect: Fayet et al. [18]	Tai Chi pilot; 40 participants; group sessions of approximately 60 min. Randomization concerned immediate versus delayed treatment, not remote versus hospital delivery.	The exploratory modality analysis detected no difference in total physical activity at month 2; an effect estimate and CI for that contrast were not reported.	Delivery mode is confounded by calendar period and the COVID-19 context; baseline data were not reported by mode.
Mixed-mode: Bundle et al. [19]	Prospective non-randomized study; axSpA online yoga n = 24, individual physiotherapy n = 24; approximately 60 min/week versus at least 30 min/week; preference-based allocation.	Numerical changes at 8–12 weeks included BASMI −0.31 versus +0.29 and BASFI −0.17 versus +0.05, online yoga versus physiotherapy.	Allocation, content, dose, and synchronous/asynchronous access differed; a delivery effect cannot be isolated.
Context: Acar et al. [20]	RCT; 60 randomized, 55 analyzed; live small-group Zoom yoga, 60 min, 3 times/week for 8 weeks, versus wait-list.	BASFI changed by −1.321 (95% CI −1.782 to −0.861) in tele-yoga and −0.178 (−0.647 to 0.291) in control; group-by-time *p* = 0.001.	Supports efficacy against no added exercise, not non-inferiority to face-to-face rehabilitation.
Context: Singh et al. [21]	Randomized trial; 109 analyzed; two 60 min guided online yoga sessions/week plus recorded materials versus standard care for 3 months.	Between-group change estimates were −0.79 (95% CI −1.30 to −0.29) for BASDAI and −0.86 (−1.59 to −0.14) for BASFI.	Mixed synchronous/asynchronous support and a non-exercise control preclude a delivery-mode comparison.
Context: Gencer et al. [22]	RCT; 68 allocated and 59 completed; live Baduanjin, 45 min twice/week for 12 weeks, versus matched-duration home exercise.	Between-group change-score tests were reported for BASFI (*p* = 0.008), sleep quality (*p* = 0.018), and lumbar flexion (*p* = 0.046); CIs were not reported.	Content and supervision differed; complete-case analysis and multiple outcomes limit inference.
Context: Sen et al. [23]	RCT; 50 allocated and 44 analyzed; identical core-stability exercise, 45 min, 3 times/week for 8 weeks, synchronous versus asynchronous.	BASFI change was 1.68 versus 0.48 points (*p* = 0.025; effect size 0.71); adherence was 95% versus 70%.	Supports a role for real-time supervision, but neither arm was face-to-face.
Context: Yentur et al. [24]	RCT; 44 randomized and 39 analyzed; live telerehabilitation for 30–40 min versus approximately 30 min selected YouTube exercise, 3 times/week for 8 weeks.	Post-treatment BASFI was 3.60 versus 3.40 (*p* = 0.799); most other between-group comparisons were also not statistically significant.	One randomized participant was not reconciled in the flow; the trial does not address face-to-face care.

Abbreviations: BASFI, Bath Ankylosing Spondylitis Functional Index; RCT, randomized controlled trial.

**Table 2:** Detailed population and intervention characteristics of the Karaca trial reported in the final doctoral thesis.

Domain	Reported Detail
Setting and flow	Single-center trial; recruitment December 2023 to March 2025. Fifty participants were randomized (25 per group), 46 were assessed post-intervention, and 40 completed six-month follow-up. The thesis used multiple imputation for an ITT analysis (25 per group) and a complete-case PP analysis (20 per group).
Age and sex	Face-to-face: 43.28 ± 10.63 years, 52% male. Telerehabilitation: 42.72 ± 11.98 years, 48% male.
Disease phenotype	Radiographic axSpA: 18/25 (72%) face-to-face and 20/25 (80%) telerehabilitation. Median diagnosis duration was 10.0 (IQR 2.25–18.5) versus 7.0 (2.5–13.0) years; symptom duration was 15.0 (9.5–25.0) versus 10.0 (8.5–18.5) years.
Baseline disease burden	Mean BASDAI was 5.52 ± 2.04 versus 4.59 ± 1.85, and median BASFI was 4.2 (IQR 2.8–5.7) versus 3.3 (2.4–5.2), face-to-face versus telerehabilitation. Although formal baseline tests were not statistically significant in the ITT sample, disease activity and several patient-reported measures were numerically worse in the face-to-face group.
Concomitant treatment	NSAIDs: 60% in both groups; conventional synthetic DMARDs: 32% versus 20%; corticosteroids: 12% versus 4%; biologic DMARDs: 32% versus 48%; other medication: 64% versus 52%, face-to-face versus telerehabilitation.
Digital eligibility	Participants or a helper had to be able to use a smartphone or computer, and participants had to have a smartphone or computer with an active internet connection. The flow diagram did not report how many otherwise eligible people were excluded or declined for technology, connectivity, home-environment, or delivery-preference reasons.
Initial assessment	A blinded assessor completed an approximately one-hour baseline assessment; patient-reported instruments were self-completed and objective measures were administered by the assessor. Post-intervention and six-month assessments were repeated in the clinic by the same blinded assessor.
Intervention and dose	Both groups received a one-hour individual education session plus a booklet, followed by 24 individual 60 min sessions (3/week for 8 weeks) with the same physiotherapist. Sessions comprised approximately 5 min warm-up, 50 min training, and 5 min cool-down. Remote sessions used Zoom or WhatsApp videoconferencing.
Progression and equipment	The program used basic, intermediate, and advanced levels and was individualized. Target intensity was Borg CR10 RPE 4–6 with 8–12 controlled, pain-free repetitions; exercises were regressed if alignment or Pilates principles could not be maintained. A mat, progressive resistance bands, and a 55 or 65 cm exercise ball were required.
Therapists and safety	The protocol was developed by two physiotherapists with clinical Pilates competence and 20 and 9 years of experience; one delivered all sessions. Adverse events were actively queried before and after sessions and observed during exercise; the program was modified when symptoms occurred.
Technical issues	Internet problems and an unsuitable home environment were listed among reasons for missed sessions, but the number and duration of technical failures were not reported.

**Table 3:** ClinicalTrials.gov version history for NCT05006690 and its relevance to interpretation.

Registry Version/Date	Status and Timing	Material Specification	Interpretive Relevance
Version 0; submitted 8 August 2021; first posted 16 August 2021	Not yet recruiting; planned start October 2021; planned completion May 2023	Estimated n = 30; three groups (telerehabilitation, face-to-face, home-based spinal stabilization); BASDAI and BASFI both primary; baseline, 8 weeks, and six months described in summary.	Prospective to the actual recruitment start. It documents the original three-arm design and two primary outcomes.
Version 1; submitted 17 July 2024	Not yet recruiting in record, but actual recruitment had begun 18 December 2023	Estimated n changed 30→50; home-based arm removed; intervention reframed as clinical Pilates; primary-outcome order reversed but BASFI and BASDAI both retained; secondary outcomes revised; six-month assessment retained in summary.	Material amendments occurred during recruitment and after preliminary results had been presented; reasons and any contemporaneous SAP were not publicly available.
Version 2; submitted 18 July 2024	Recruiting; actual start entered as 18 December 2023; estimated completion August 2025	No outcome change from version 1; contact/location and recruitment status updated.	Confirms that version 1 amendments were posted after recruitment had begun.
Version 3; submitted 19 August 2025; posted 24 August 2025	Completed; actual completion 19 August 2025	Actual n = 50; no further outcome changes; primary outcomes remained BASFI and BASDAI.	Current record confirms enrollment but does not replace the prospectively available versions for RoB 2 Domain 5.
Thesis, 2025	Recruitment December 2023–Mar 2025	BASFI described as sole primary outcome; BASDAI secondary; ITT with multiple imputation plus PP analyses.	Difference in designation, absence of a public SAP, and multiple eligible analyses/time points create uncertainty, not proof, about result selection.

**Table 4:** Risk-of-bias summary for the prespecified study results. Assessments were completed by one review author and were not independently duplicated.

Study and Result	Tool	Domain Judgments	Overall
Karaca: BASFI, 8 weeks [14,15,16,17]	RoB 2	D1 L; D2 L; D3 SC; D4 SC; D5 SC	Some concerns
Karaca: BASFI, 6 months [14,15,16,17]	RoB 2	D1 L; D2 L; D3 SC; D4 SC; D5 SC	Some concerns
Acar: BASFI, 8 weeks [20]	RoB 2	D1 L; D2 L; D3 L; D4 SC; D5 L	Some concerns
Singh: BASFI, 3 months [21]	RoB 2	D1 L; D2 H; D3 H; D4 SC; D5 SC	High
Gencer: BASFI, 12 weeks [22]	RoB 2	D1 SC; D2 SC; D3 SC; D4 SC; D5 SC	High *
Sen: BASFI, 8 weeks [23]	RoB 2	D1 L; D2 H; D3 H; D4 SC; D5 SC	High
Yentur: BASFI, 8 weeks [24]	RoB 2	D1 SC; D2 SC; D3 H; D4 SC; D5 SC	High
Fayet: total physical activity, remote vs hospital [18]	ROBINS-I	D1 C; D2 S; D3 L; D4 S; D5 S; D6 S; D7 S	Critical
Bundle: BASFI, online yoga vs physiotherapy [19]	ROBINS-I	D1 S; D2 S; D3 S; D4 S; D5 M; D6 S; D7 S	Serious

RoB 2 domains: D1, randomization; D2, deviations from intended intervention; D3, missing outcome data; D4, outcome measurement; D5, selection of the reported result. RoB 2 judgments: L, low risk; SC, some concerns; H, high risk. ROBINS-I domains: D1, confounding; D2, selection of participants; D3, classification of interventions; D4, deviations from intended interventions; D5, missing data; D6, measurement of outcomes; D7, selection of the reported result. ROBINS-I judgments: L, low; M, moderate; S, serious; C, critical. * For Gencer, concerns in multiple domains jointly lowered confidence; no single domain was independently sufficient for a high-risk judgment.

**Table 5:** Methodological priorities for direct comparative trials.

Domain	Recommended Design Feature	Reason
Comparative estimand	Non-inferiority hypothesis with a prespecified margin justified clinically and statistically; interpret the CI against the margin.	Matches the substitution question and prevents interpreting a non-significant superiority test as non-inferiority.
Intervention control	Identical exercise content, dose, therapist contact, and progression rules; delivery mode as the intended difference.	Isolates the effect of remote versus face-to-face delivery.
Sample and setting	Adequately powered multicenter recruitment with broad disease activity and digital-literacy profiles.	Improves precision and generalizability.
Bias control	Concealed allocation, blinded outcome assessment, prespecified missing-data methods, and both intention-to-treat and per-protocol analyses.	Supports valid non-inferiority inference.
Outcomes	Justified primary endpoint (e.g., BASFI, disease activity, mobility, or patient-centered outcome), confidence intervals, multiplicity control, and clinically important change.	Separates statistical from clinical relevance and aligns the endpoint with the treatment goal.
Durability	Follow-up extending pragmatically to 6–12 months, with adherence and maintenance of effect measured over time.	Tests whether access advantages translate into sustained participation and benefit.
Implementation	Session attendance, technical failures, adverse events, satisfaction, access barriers, costs, and healthcare use.	Characterizes observed harms, equity, and scalability under prespecified monitoring.

## Data Availability

No new individual-participant data were generated. Complete database-specific strategies are supplied in Supplementary Section S1; the record-level screening and deduplication log is supplied as Supplementary Data S1; the full-text exclusion record and language-handling description are supplied in Supplementary Section S2; the source-referenced and independently verified Karaca thesis extraction is supplied in Supplementary Section S4; the ClinicalTrials.gov version history is supplied in Supplementary Section S5; and the signaling-question and domain-level risk-of-bias rationales are supplied in Supplementary Section S6.

## Supplementary Materials

The following supporting information can be downloaded at https://www.mdpi.com/article/10.3390/healthcare14203384/s1.

## Abbreviations

The following abbreviations are used in this manuscript:

AS	Ankylosing spondylitis
axSpA	Axial spondyloarthritis
BASDAI	Bath Ankylosing Spondylitis Disease Activity Index
BASFI	Bath Ankylosing Spondylitis Functional Index
BASMI	Bath Ankylosing Spondylitis Metrology Index
CI	Confidence interval
ITT	Intention-to-treat
PP	Per-protocol
RCT	Randomized controlled trial
RoB 2	Risk of Bias 2 tool for randomized trials
ROBINS-I	Risk Of Bias In Non-randomized Studies-of-Interventions
RPE	Rating of perceived exertion
TR	Telerehabilitation
