# Supplementary material for: Synchronous Physical Telerehabilitation Versus Face-to-Face Rehabilitation in Axial Spondyloarthritis: A Focused Narrative Review
Source: Healthcare (Basel). 2026 Oct 11;14(20):3384. doi: 10.3390/healthcare14203384 (PMC13704107; doi:10.3390/healthcare14203384)
Supplement: Supplementary file 1 [file healthcare-14-03384-s001.zip › healthcare-4525755 supplementary materials.pdf]

Supplementary Materials

Synchronous physical telerehabilitation versus face-to-face rehabilitation in axial spondyloarthritis

S1. Search strategies and search log

The five databases reported in the manuscript were rerun from inception on 9 September 2026 without date, language, or study-design restrictions. The complete strings below reproduce the strategies used. Export files were retained for PubMed, CENTRAL, PEDro, Scopus, and Web of Science. ClinicalTrials.gov, linked-report, reference-list, and forward-citation searches were completed on 11 September 2026. The two-day difference reflects sequential processing after receipt of the database exports and could only add records to the evidence set.

S1.1 PubMed/MEDLINE

Platform/status: PubMed (National Library of Medicine). Search date: 9 September 2026; yield: 30 records.

```
("Spondylitis, Ankylosing"[Mesh] OR "ankylosing spondylitis"[tiab] OR "axial spondyloarthritis"[tiab] OR axSpA[tiab]) AND ("Telerehabilitation"[tiab] OR "tele-rehabilitation"[tiab] OR telerehab*[tiab] OR "Telemedicine"[Mesh] OR telemedicine[tiab] OR telehealth[tiab] OR "remote rehabilitation"[tiab] OR "remote physiotherapy"[tiab] OR "online physiotherapy"[tiab] OR "online exercise"[tiab] OR "web-based exercise"[tiab] OR "internet-based exercise"[tiab] OR "app-based"[tiab] OR "mobile app*"[tiab] OR "video-based"[tiab] OR "virtual rehabilitation"[tiab] OR "tele-yoga"[tiab] OR "tele yoga"[tiab]) AND ("Physical Therapy Modalities"[Mesh] OR "Exercise Therapy"[Mesh] OR "Rehabilitation"[Mesh] OR physiotherapy[tiab] OR "physical therapy"[tiab] OR rehabilitation[tiab] OR exercise[tiab] OR stretching[tiab] OR strengthening[tiab] OR yoga[tiab])
```

Export retained as pubmed_2026_09_09.nbib. The export does not itself preserve the query history; retain the dated strategy document or a search-history screenshot with the submission records.

S1.2 Cochrane CENTRAL

Platform/status: Cochrane Library, CENTRAL trials search. Search date: 9 September 2026; yield: 10 records.

```
("ankylosing spondylitis" OR "axial spondyloarthritis" OR axSpA) AND (telerehabilitation OR "tele-rehabilitation" OR telerehab* OR telemedicine OR telehealth OR "remote rehabilitation" OR "remote physiotherapy" OR "online physiotherapy" OR "online exercise" OR "web-based exercise" OR "internet-based exercise" OR "app-based" OR (mobile NEXT app*) OR "video-based" OR "virtual rehabilitation" OR "tele-yoga" OR "tele yoga") AND (physiotherapy OR "physical therapy" OR rehabilitation OR "exercise therapy" OR exercise OR stretching OR strengthening OR yoga)
```

Export retained as cochrane_2026_09_09.ris. The records are trial/article records rather than the Cochrane Review records present in the legacy export.

S1.3 Scopus

Platform/status: Scopus (Elsevier). Search date: 9 September 2026; yield: 98 records.

```
TITLE-ABS-KEY (("ankylosing spondylitis" OR "axial spondyloarthritis" OR axSpA) AND (telerehabilitation OR telemedicine OR telehealth OR remote OR online OR web-based OR app-based) AND (physiotherapy OR rehabilitation OR exercise OR yoga))
```

Export retained as scopus_2026_09_09.ris, including citations, abstracts, identifiers, and the export date.

S1.4 Web of Science Core Collection

Platform/status: Web of Science Core Collection. Search date: 9 September 2026; yield: 72 records. The RIS export identifies Web of Science records but does not preserve the individual citation indexes selected.

```
TS=(("ankylosing spondylitis" OR "axial spondyloarthritis" OR axSpA) AND (telerehabilitation OR telemedicine OR telehealth OR remote OR online OR web-based OR app-based) AND (physiotherapy OR rehabilitation OR exercise OR yoga))
```

Export retained as wos_2026_09_09.ris. The absence of index-level search-history metadata is reported as a reproducibility limitation.

S1.5 PEDro

Platform/status: PEDro. Search date: 9 September 2026; supplied workbook: four unique records.

Run separately:
1. ankylosing spondylitis telemedicine
2. ankylosing spondylitis telerehabilitation
3. ankylosing spondylitis exercise online
4. ankylosing spondylitis physiotherapy remote

The workbook pedro_2026_09_09.xlsx contains four unique records across the four simple searches. Separate raw yields and query-to-record mappings were not preserved; the aggregate PEDro yield is therefore reported transparently.

S1.6 ClinicalTrials.gov and citation searching

Platform/status: ClinicalTrials.gov registry search, linked-report tracing, backward reference-list checks, and OpenAlex forward-citation searches. Final supplementary-search date: 11 September 2026.

```
AREA[ConditionSearch]("ankylosing spondylitis" OR "axial spondyloarthritis" OR axSpA) AND (telerehabilitation OR "tele-rehabilitation" OR "remote rehabilitation" OR "remote physiotherapy" OR "online physiotherapy" OR "web-based physiotherapy" OR "remote exercise" OR "online exercise" OR "video-based exercise" OR "tele-yoga" OR "tele yoga" OR videoconference)
```

ClinicalTrials.gov was searched with the exact fielded query below and returned 10 records. References of retained outcome reports were checked for linked or additional comparative studies. Forward citations to nine seed reports were retrieved in OpenAlex, deduplicated by work identifier/DOI, and screened (50 unique citing works). No additional completed direct trial was retained. Two citation-search records were excluded as clearly out of scope: a phase I evaluation of the asynchronous ExerciseRx platform (10.1186/s42358-026-00519-1) and a secondary review/proposed rehabilitation protocol (10.4103/jdmimsu.jdmimsu_291_24). One new ongoing contextual registry, NCT07503327, had no face-to-face arm and no results.

S1.7 Search log

Source	Platform	Exact date	Limits	Yield	Export/history file	Status
PubMed/MEDLINE	PubMed/NLM	9 Sep 2026	None	30	pubmed_2026_09_09.nbib	Complete
CENTRAL	Cochrane Library/CENTRAL	9 Sep 2026	None stated	10	cochrane_2026_09_09.ris	Complete
PEDro	PEDro	9 Sep 2026	None	4 unique	pedro_2026_09_09.xlsx	Aggregate yield
Scopus	Elsevier Scopus	9 Sep 2026	None	98	scopus_2026_09_09.ris	Complete
Web of Science	Core Collection; individual indexes not retained	9 Sep 2026	None	72	wos_2026_09_09.ris	Index metadata unavailable

Source	Platform	Exact date	Limits	Yield	Export/history file	Status
ClinicalTrials.gov	NLM	11 Sep 2026	None	10	clinicaltrials_gov_10_records_screening.csv	Complete
Backward references	Reference lists of retained outcome reports	11 Sep 2026	Eligible seed reports	No additional report retained	Narrative audit	Complete; cited-reference count not recorded
Forward citations	OpenAlex; nine seed reports	11 Sep 2026	None	50 unique citing works	openalex_50_citing_works_comparison.csv	Complete

S2. Study selection record

The 214 database records were deduplicated by prioritizing exact DOI and PMID matches, followed by normalized exact-title matching and manual comparison of non-identical title variants. Three pairs of database-specific conference-record variants were merged after comparison. Two different Korean guideline publications with the same title but different DOIs and journals were retained as separate bibliographic records. This process removed 91 duplicate copies and left 123 unique records. Machine assistance was limited to deduplication, reconciliation of bibliographic identifiers and title variants, record organization, and assembly of the audit trail; no record was automatically included or excluded. All final eligibility decisions and reason codes were determined by human reviewers. One hundred eleven records were excluded at title/abstract screening, 12 reports were retrieved and assessed in full or expanded form, one was excluded, and 11 reports representing eight studies were selected from the databases. The ClinicalTrials.gov record and doctoral thesis linked to the Karaca trial were obtained separately through supplementary searches and were not counted among those 11 database-selected reports. Initial study-selection decisions were made by one review author in Rayyan. During revision, a second review author (M.D.G.) independently verified the eligibility decisions and exclusion coding for all 123 records against the stated evidence categories. No discrepancies were identified, so consensus adjudication was not required. This was retrospective verification rather than prospective parallel dual screening. The complete 123-record log, including database provenance and reasons, is supplied as Supplementary Data S1.

No language filter was applied. All 123 records were screened using the titles, abstracts, and indexing metadata supplied by the databases, including English-language titles or abstracts where available for non-English publications. No record was excluded solely because of language. Records that were clearly ineligible from this information were excluded for the substantive reasons recorded in Supplementary Data S1; when the information was insufficient to exclude a record confidently, the report was advanced for fuller assessment irrespective of language. The Karaca doctoral thesis was the only potentially eligible non-English source for which full-text translation was required. Its Turkish full text was translated and independently checked as described in Supplementary Section S4.

Flow item	Count	Audit note
Database records identified	214	30 PubMed + 10 CENTRAL + 4 PEDro + 98 Scopus + 72 Web of Science
Duplicate copies removed	91	DOI/PMID/title matching plus comparison of title variants

Flow item	Count	Audit note
Unique records screened	123	Complete record-level log supplied as Supplementary Data S1
Records excluded at title/abstract	111	Reasons retained in the record-level log
Reports sought/retrieved	12 / 12	Full or expanded report obtained for each
Full-text reports excluded	1	Mixed rheumatic sample; only 5 AS participants; no analyzable axSpA-specific comparison; no face-to-face comparator
Reports retained from database screening	11	Representing 8 studies; the linked ClinicalTrials.gov record and doctoral thesis were obtained separately and are not counted here
Direct evidence	1 study	Karaca trial; 2 database-selected abstracts plus a separately obtained ClinicalTrials.gov record and doctoral thesis
Indirect/mixed-mode evidence	2 studies	Fayet and Bundle
Illustrative contextual evidence	5 studies	Acar, Singh, Gencer, Sen, and Yentur

Study-identification and selection record

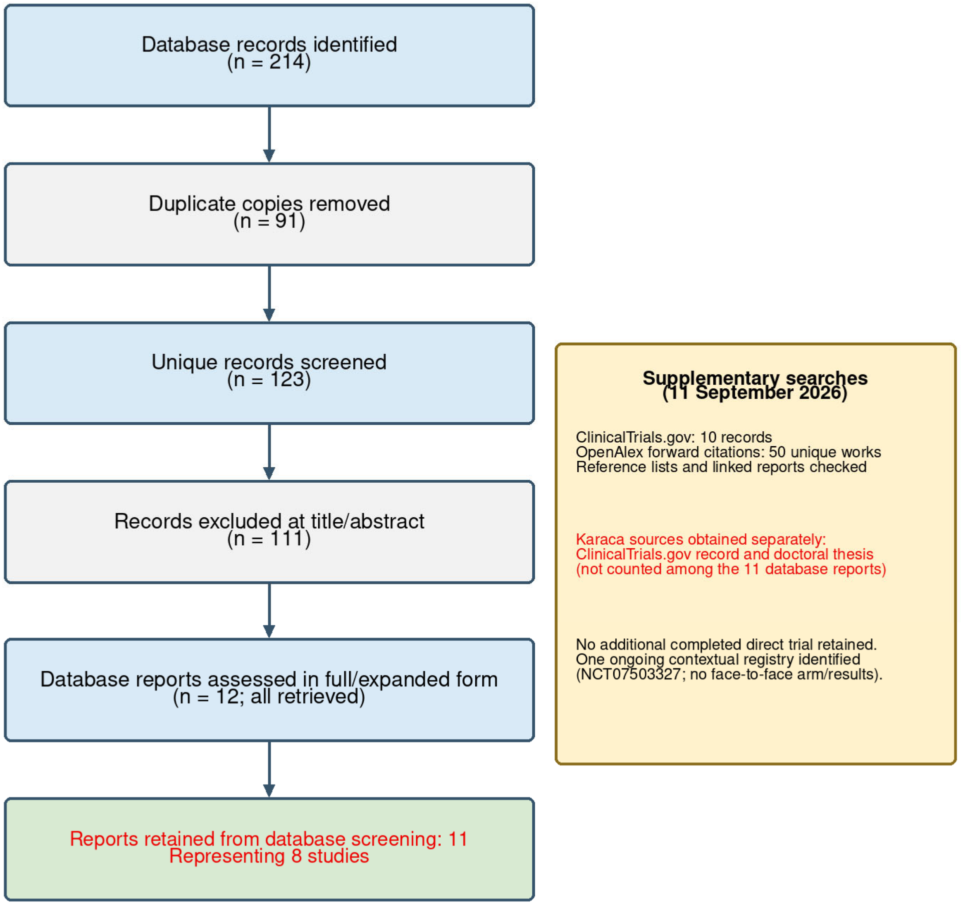

Full-text exclusion (n = 1): mixed rheumatic-disease sample; only five participants with AS, no analyzable axSpA-specific comparative effect, and no face-to-face comparator.

Figure S1. Study-identification and selection flow for the 9 September 2026 database update and 11 September 2026 supplementary searches.

S2.1 Full-text and scope-near exclusion record

Citation/registry	Stage	Report obtained?	Primary reason	Category	Route	Final
U082; Bağlan Yentür et al.; doi:10.21653/tjpr.1195457	Full text	Yes	Only 5 AS participants; no analyzable axSpA-specific contrast; no face-to-face comparator	Contextual	Database screening	Exclude
Stovall et al.; doi:10.1186/s42358-026-00519-1	Citation title/abstract	Abstract/record	Phase I evaluation of an asynchronous digital exercise platform; no eligible delivery comparison	Contextual	Forward citation	Exclude
Bhakaney and Tiwari; doi:10.4103/jdmimsu.jdmimsu_291_24	Citation title/record	Record	Secondary review with a proposed rehabilitation protocol; not a primary outcome study	Contextual	Forward citation	Exclude
NCT07503327	Registry	Registry record	Recruiting; no face-to-face arm and no results	Ongoing contextual	Registry search	Exclude from completed-study synthesis

S3. Evidence categories and contextual-selection principles

Category	Eligibility rule	Permitted interpretation
Direct	Adults with axSpA/AS; live videoconference rehabilitation; supervised face-to-face comparator; same intended program/content and comparable dose.	Supports the delivery-mode question, subject to precision and bias.
Indirect	Remote and in-person delivery compared, but allocation to delivery mode not randomized or delivery is tied to calendar/context.	Cannot isolate delivery mode; useful for hypothesis generation.
Mixed-mode	Live and prerecorded remote content mixed, or intervention content/dose differs between remote and comparator arms.	Addresses a package comparison, not delivery-mode substitution.
Illustrative contextual	AxSpA/AS-specific results; live remote component; comparator informative for feasibility, attendance, adverse-event reporting, or real-time supervision; results available.	Selected examples only. No claim of completeness, balance, prevalence, comparative safety, or substitution.

S4. Karaca thesis extraction and source-verification table

Independent bilingual verification was completed in September 2026. An independent authorized translator proficient in Turkish and English compared the Turkish original with this table and the thesis-derived manuscript statements for printed pages 21–24, 26–30, 37–41, 43–44, 80, 82, and 104–105 (PDF pages 38–41, 43–47, 54–58, 60–61, 97, 99, and 121–122). The check covered outcome labels, methodological descriptions, groups, denominators, analysis

populations, time points, statistical tests, and every numerical value. The translator confirmed the accuracy of the translated and transcribed information; any wording corrections identified during verification were incorporated. The authors retain the written verification record.

Original Turkish label	English translation	Source (printed/PDF)	Denominator	Population	Time	Method	Extracted item/estimate
Araştırmanın Tipi	Study type	p. 21 (PDF p. 38), §3.1	50 randomized	Trial-level	—	Design description	Randomized, single-blind, parallel-group clinical trial
Dahil Edilme Kriterleri	Inclusion criteria	p. 22 (PDF p. 39), §3.4.2	Candidates	Eligibility	Pre-randomization	Eligibility checklist	Participant or helper able to use smartphone/computer; participant required a smartphone or computer with an active internet connection
Örneklem Boyutu Hesaplaması	Sample-size calculation	p. 22 (PDF p. 39), §3.4.4	Planned n = 50	Design	Pre-trial	Repeated-measures within–between interaction; f = 0.211; α = 0.05; power = 80%; +20% attrition	Required n = 38; planned n = 50
Araştırma Tasarımı — Prosedür	Randomization and concealment	pp. 23–24 (PDF pp. 40–41), §3.5.1	50 randomized	Trial-level	After baseline	random.org sequence; 50 sequential identical opaque sealed envelopes	1:1 allocation; envelope opened by treating physiotherapist after baseline
Egzersiz Programı	Intervention dose and delivery	pp. 23–29 (PDF pp. 40–46), §3.5	25/group	Assigned groups	8 weeks	24 individual 60-min sessions; 3/week; same physiotherapist; matched content	Remote by Zoom/WhatsApp versus in-clinic; 5-min warm-up, 50-min exercise, 5-min cool-down; individualized progression
Egzersiz Programı — şiddet/ekipman	Intensity, progression, and equipment	pp. 26–29 (PDF pp. 43–46)	25/group	Assigned groups	8 weeks	Borg CR10 RPE 4–6; 8–12 pain-free controlled repetitions; basic/intermediate/advanced levels	Mat, resistance bands, and 55-/65-cm exercise ball; regression when alignment or Pilates principles could not be maintained
BASFI	Primary outcome designation	p. 30 (PDF p. 47), §3.6.1	50 randomized	Outcome	T1/T2/T3	BASFI	Thesis identifies BASFI as primary; BASDAI is secondary
İstatistiksel Analiz	Statistical analysis	pp. 37–38 (PDF pp. 54–55), §3.7	ITT n = 25/group; PP n = 20/group	ITT and PP	T1 baseline; T2 month 2; T3 month 8	Friedman plus Wilcoxon within groups; Mann–Whitney U on change scores between groups; multiple imputation for ITT	Bonferroni-adjusted significance threshold α = 0.017 for three pairwise time contrasts; the p values themselves were not adjusted
Şekil 4.1. CONSORT akış diyagramı	CONSORT flow diagram	p. 39 (PDF p. 56), Figure 4.1	189 assessed; 50 randomized	Observed flow	T2/T3	Counts	139 excluded (62 ineligible; 77 declined); 25/25 randomized; 46 assessed at T2; 40 at T3; no technology-specific exclusion/refusal breakdown
Bulgular — katılımcı akışı	Participant-flow narrative	p. 39 (PDF p. 56)	50 randomized	Observed flow	T2/T3	Narrative versus diagram	Narrative states six left before treatment ended, whereas Figure 4.1 shows four lost before T2 and six additional losses before T3. The manuscript follows the diagram: 50 → 46 → 40.
Tablo 4.1	Demographic and treatment characteristics	p. 40 (PDF p. 57), Table 4.1	ITT n = 25/group	ITT	Baseline	Descriptive statistics	Age, sex, phenotype, disease/symptom duration, and concomitant medication used in Manuscript Table 2
Tablo 4.2	Baseline BASFI/BASDAI	p. 41 (PDF p. 58), Table 4.2	ITT n = 25/group	ITT	Baseline	Descriptive statistics	Face-to-face BASDAI 5.52 ± 2.04 and BASFI 4.2 (2.8–5.7); remote BASDAI 4.59 ± 1.85 and BASFI 3.3 (2.4–5.2)
Tablo 4.5	BASFI descriptive data	p. 43 (PDF p. 60), Table 4.5	ITT n = 25/group	ITT	T1/T2/T3	Median (IQR)	Face-to-face: 4.2 (2.8–5.7), 1.0 (0.5–2.0), 2.0 (0.7–2.7); remote: 3.3 (2.4–5.2), 0.7 (0.0–1.5), 1.0 (0.3–2.0)
Tablo 4.5	BASFI descriptive data	p. 43 (PDF p. 60), Table 4.5	PP n = 20/group	PP complete cases	T1/T2/T3	Median (IQR)	Face-to-face: 4.5 (3.6–5.7), 1.4 (0.4–2.1), 2.0 (0.5–3.4); remote: 3.4 (2.1–4.9), 0.8 (0.1–1.5), 1.0 (0.3–1.9)

Original Turkish label	English translation	Source (printed/PDF)	Denominator	Population	Time	Method	Extracted item/estimate
Tablo 4.7	Between-group BASFI change	p. 44 (PDF p. 61), Table 4.7	ITT n = 25/group	ITT	ΔT1–T2	Mann–Whitney U; α = 0.017	Face-to-face 3.0 (1.1–4.3) vs remote 2.5 (1.8–3.4); p = 0.587
Tablo 4.7	Between-group BASFI change	p. 44 (PDF p. 61), Table 4.7	ITT n = 25/group	ITT	ΔT1–T3	Mann–Whitney U; α = 0.017	Face-to-face 2.0 (1.0–3.7) vs remote 2.0 (1.1–3.2); p = 0.838
Tablo 4.7	Between-group BASFI change	p. 44 (PDF p. 61), Table 4.7	ITT n = 25/group	ITT	ΔT2–T3	Mann–Whitney U; α = 0.017	Face-to-face −0.7 (−1.5 to 0.3) vs remote −0.5 (−1.0 to 0.1); p = 0.521
Tablo 4.7	Between-group BASFI change	p. 44 (PDF p. 61), Table 4.7	PP n = 20/group	PP complete cases	ΔT1–T2	Mann–Whitney U; α = 0.017	Face-to-face 3.0 (1.1–4.6) vs remote 2.5 (1.6–3.4); p = 0.490
Tablo 4.7	Between-group BASFI change	p. 44 (PDF p. 61), Table 4.7	PP n = 20/group	PP complete cases	ΔT1–T3	Mann–Whitney U; α = 0.017	Face-to-face 1.9 (0.9–4.1) vs remote 2.1 (0.8–3.1); p = 0.989
Tablo 4.7	Between-group BASFI change	p. 44 (PDF p. 61), Table 4.7	PP n = 20/group	PP complete cases	ΔT2–T3	Mann–Whitney U; α = 0.017	Face-to-face −0.4 (−1.5 to 0.2) vs remote −0.5 (−0.9 to 0.2); p = 0.473
Hastaların Tedavi Uyumu — Bulgular	Attendance/adherence findings	p. 80 (PDF p. 97), §4.3.5	46 completers; 50 randomized	Completer and all-randomized	24-session intervention	Sessions attended / 24	Completers: 98.2% face-to-face, 98.4% remote; all randomized: 93.8% and 94.0%; exact between-group p value not stated
İstenmeyen Olaylara İlişkin Bulgular	Adverse-event findings	p. 82 (PDF p. 99), §4.3.6	50 randomized	Observed participants	Intervention and follow-up	Descriptive ascertainment	Five transient pain/cramp events (3 vs 2); two serious events during treatment and one during follow-up judged unrelated; no intervention-related serious adverse event reported
Çalışmanın Limitasyonları	Study limitations	pp. 104–105 (PDF pp. 121–122), §5.8	Trial-level	—	—	Author-reported limitations	Single center; digital eligibility; participants/therapist unblinded; follow-up attrition; no inflammatory biomarkers; limited power for some secondary analyses

S5. ClinicalTrials.gov history — NCT05006690

Version	Submitted / posted	Status/start	Enrollment	Arms/intervention	Primary outcomes	Follow-up	Interpretive note
0	8 Aug 2021 / 16 Aug 2021	Not yet recruiting	30 estimated	Three arms: tele, face-to-face, home-based	BASDAI + BASFI	Baseline, 8 weeks, 6 months in summary	Prospective to actual recruitment
1	17 Jul 2024	Not yet recruiting	50 estimated	Two arms; home arm removed; clinical Pilates specified	BASFI + BASDAI (same outcomes, reversed order)	Six months retained in summary; outcome fields describe 8 weeks	Posted after recruitment began; secondary outcomes revised
2	18 Jul 2024	Recruiting; actual start 18 Dec 2023	50 estimated	No further arm change	No change	No change	Status/contact update
3	19 Aug 2025 / 24 Aug 2025	Completed	50 actual	No change	No change	No change	Completion/enrollment update

Source: ClinicalTrials.gov historical API/Record History for NCT05006690, retrieved 7 September 2026 and rechecked 11 September 2026. Both BASDAI and BASFI were primary outcomes from version 0; a material redesign occurred during recruitment. Domain 5 therefore acknowledges both the prospective co-primary outcomes and the absence of a public, dated statistical analysis plan.

S6. Risk-of-bias assessments

Outcome rule: the between-group BASFI result at the principal end-of-intervention time point was assessed for every randomized study; Karaca BASFI at six months was additionally assessed because durability was central to the review question. The RoB 2 estimand was the effect of assignment to intervention. The original 2016 ROBINS-I tool was used for the two non-randomized delivery-mode contrasts. One review author completed the assessments during revision; they were not independently duplicated and disagreement resolution was not applicable. The tables below record the source-based answers that drove each domain judgment. Judgments were derived using the RoB 2 algorithm and the overall ROBINS-I rule rather than by counting domains.

S6.1 RoB 2 — Karaca BASFI at 8 weeks and 6 months

Result	Domain	Key signaling answers and source-based rationale	Judgment
Both results	D1 Randomization	Random sequence: yes (random.org, generated by a researcher outside the trial). Concealment: yes (50 sequentially numbered, identical, opaque, sealed envelopes opened after baseline). Baseline imbalance suggesting a randomization problem: no clear pattern. Thesis pp. 23 and 40–42; registry NCT05006690.	Low
Both results	D2 Deviations	Participants and the treating physiotherapist knew delivery mode, but both arms received the same therapist, individualized Pilates content, frequency, duration, patient education, and usual medical care. No trial-context deviation likely to affect BASFI was identified. The primary analysis retained all randomized participants using multiple imputation and analyzed them in their assigned groups.	Low
8 weeks	D3 Missing data	Observed BASFI was available for 46/50 participants. Multiple imputation restored n = 25 per group, but the imputation model, variables, diagnostics, and sensitivity analyses were not reported. Health events, biologic initiation, and low adherence could be related to functional status; bias cannot be excluded, although ITT and complete-case results were broadly consistent.	Some concerns
6 months	D3 Missing data	Observed BASFI was available for 40/50 participants. Multiple imputation restored n = 25 per group, but model specification and sensitivity analyses were not reported. The 20% missingness and reasons including scheduling, refusal, and health events leave plausible dependence on true function despite broadly consistent ITT and complete-case results.	Some concerns
Both results	D4 Outcome measurement	BASFI is an appropriate validated self-report measure and was administered in the same way to both groups. Participants necessarily knew delivery mode. Knowledge could influence perceived-function reporting, and assessor blinding cannot remove that possibility; however, the matched active interventions provide no clear evidence that reporting was likely influenced differently between groups.	Some concerns
Both results	D5 Reported-result selection	No dated public statistical analysis plan was located. Registry version 0 prospectively listed BASDAI and BASFI as co-primary; version 1, posted during recruitment, retained both but changed the arm structure, enrollment, intervention description, and secondary outcomes. The thesis later called BASFI the sole primary outcome. Multiple time points and analytic populations were available, but the record does not prove selection based on results.	Some concerns

Result	Domain	Key signaling answers and source-based rationale	Judgment
8 weeks	Overall	At least one domain raised some concerns and no domain was judged high risk. The combination did not meet the RoB 2 threshold for high risk because randomization was secure, all randomized participants were included in the ITT analysis, and the main uncertainties were incompletely reported rather than demonstrably result-determining.	Some concerns
6 months	Overall	The larger amount of missing observed data increases uncertainty, but multiple imputation and a consistent complete-case analysis prevent a definite high-risk judgment. Together with self-report and reporting-selection uncertainty, the result remains at some concerns.	Some concerns

S6.2 RoB 2 — remaining randomized studies

Study/result	Domain	Key signaling answers and source-based rationale	Judgment
Acar; BASFI; 8 weeks	D1 Randomization	Random Allocation software generated a block sequence; sequentially numbered opaque sealed envelopes were used; allocation followed baseline assessment; no important baseline imbalance was reported.	Low
Acar; BASFI; 8 weeks	D2 Deviations	Participants/instructors were aware, but no trial-context deviation was identified; make-up sessions were permitted and assigned groups were maintained. Post-randomization outcome absence is considered in D3.	Low
Acar; BASFI; 8 weeks	D3 Missing data	BASFI was available for 55/60 (28/30 tele-yoga; 27/30 control). Reported reasons were COVID-19, medication change, or business travel and were judged unlikely to depend on true BASFI; no marked arm imbalance was present.	Low
Acar; BASFI; 8 weeks	D4 Outcome measurement	BASFI was valid and measured identically, but it was self-reported by participants who knew assignment. Influence was possible; no direct evidence showed that the assessment was likely distorted between groups.	Some concerns
Acar; BASFI; 8 weeks	D5 Reported-result selection	BASFI was explicitly identified as the primary outcome, the trial was registered (NCT04803383), one post-treatment time point was used, and the prespecified mixed-ANOVA interaction was reported. No eligible measurement or analysis appears omitted on the available record.	Low
Acar; BASFI; 8 weeks	Overall	Only outcome-measurement awareness raised some concerns; no domain was high risk.	Some concerns
Singh; BASFI; 3 months	D1 Randomization	An independent statistician generated an unpredictable computer sequence with randomizer.org and held sequentially numbered opaque sealed envelopes. Baseline differences did not suggest randomization failure.	Low
Singh; BASFI; 3 months	D2 Deviations	Participants and therapists were aware. The report states that only participants attending at least 70% of yoga classes were eligible for analysis. This post-randomization adherence restriction is incompatible with the assignment-effect estimand and could materially favor adherers.	High
Singh; BASFI; 3 months	D3 Missing data	Outcome data were analyzed for 109/122 participants, with more loss in control (9) than yoga (4). Reasons included irregular attendance/time constraints, nonresponse to repeated calls, and COVID-19; all participants with missing outcomes were excluded. Differential missingness could depend on true function.	High
Singh; BASFI; 3 months	D4 Outcome measurement	BASFI was appropriate and measured similarly. Although outcome raters were described as blinded, BASFI was self-reported by unblinded participants; influence was possible but not demonstrated as likely.	Some concerns
Singh; BASFI; 3 months	D5 Reported-result selection	The trial was prospectively registered (CTRI/2020/08/027215) and a protocol was published, but BASFI was one of several primary outcomes and several within/between-group analytic routes were available. No dated detailed SAP was located, and numerical/reporting inconsistencies increase uncertainty.	Some concerns
Singh; BASFI; 3 months	Overall	High risk in deviations and missing-data domains determines the overall judgment.	High
Gencer; BASFI; 12 weeks	D1 Randomization	The report states block randomization stratified by age and sex, but does not describe sequence generation or allocation concealment. Baseline outcome distributions were broadly similar.	Some concerns
Gencer; BASFI; 12 weeks	D2 Deviations	Participants/providers were unblinded; adherence and trial-context deviations were not reported in enough detail, and only completers were analyzed. It is unclear whether deviations or analytic exclusions could affect the assignment effect.	Some concerns
Gencer; BASFI; 12 weeks	D3 Missing data	Fifty-nine of 68 randomized participants were analyzed (five intervention; four control withdrew). Reasons were summarized only as various and no imputation or sensitivity analysis was reported, so dependence on true BASFI cannot be excluded.	Some concerns
Gencer; BASFI; 12 weeks	D4 Outcome measurement	BASFI was a valid self-report measure; participants were unblinded and all assessments were performed by the same physiotherapist. Measurement was consistent, but awareness could influence reports.	Some concerns
Gencer; BASFI; 12 weeks	D5 Reported-result selection	The report cites NCT05771376, but no dated detailed analysis plan was available for assessment. Numerous outcomes and within-group, post-treatment, and change-score analyses were possible; the reported BASFI change contrast therefore has unresolved selection risk.	Some concerns

Study/result	Domain	Key signaling answers and source-based rationale	Judgment
Gencer; BASFI; 12 weeks	Overall	Some concerns across all five domains jointly substantially lower confidence, meeting the RoB 2 rule for high risk even though no single domain was independently judged high.	High
Sen; BASFI; 8 weeks	D1 Randomization	An independent researcher generated a computer sequence; assignments were placed in opaque envelopes and opened after eligibility/baseline procedures. No baseline imbalance suggested failure.	Low
Sen; BASFI; 8 weeks	D2 Deviations	Participants/providers were aware. Three synchronous and three asynchronous participants were excluded after randomization; inability to attend or perform exercises regularly was tied to receipt/adherence. The complete-case analysis did not preserve the assignment effect and could materially alter the contrast.	High
Sen; BASFI; 8 weeks	D3 Missing data	BASFI was available for 44/50 randomized participants. In the asynchronous arm, all three losses were unable to exercise regularly and missed post-treatment assessment; missing outcomes were therefore plausibly related to both intervention adherence and true improvement. No imputation/sensitivity analysis was reported.	High
Sen; BASFI; 8 weeks	D4 Outcome measurement	BASFI was appropriate and administered similarly, but participants knew whether exercise was live or prerecorded. A blinded assessor of physical tests does not eliminate possible influence on participant-reported BASFI.	Some concerns
Sen; BASFI; 8 weeks	D5 Reported-result selection	The trial was prospectively registered (NCT06000072), but numerous outcomes and parametric/nonparametric analytic choices were available and no dated detailed SAP was located. The BASFI result was reported, but selection among analyses cannot be ruled out.	Some concerns
Sen; BASFI; 8 weeks	Overall	High risk in deviations and missing-data domains determines the overall judgment.	High
Yentur; BASFI; 8 weeks	D1 Randomization	Random.org was used, but sequence restrictions and allocation concealment were not described. Baseline outcomes were broadly similar; concealment remains uncertain.	Some concerns
Yentur; BASFI; 8 weeks	D2 Deviations	Participants/providers were unblinded and monitoring differed by arm. Only post-treatment completers were analyzed, with withdrawals related to continuation; no ITT or sensitivity analysis was reported.	Some concerns
Yentur; BASFI; 8 weeks	D3 Missing data	BASFI was available for 39/44 randomized participants (21 telerehabilitation; 18 YouTube). One telerehabilitation participant had surgery, three YouTube participants declined continuation, and one randomized participant was not clearly accounted for. The differential, partly unexplained loss could depend on true outcome.	High
Yentur; BASFI; 8 weeks	D4 Outcome measurement	BASFI was valid and measured consistently, but self-reported by participants aware of live-supervised versus YouTube delivery. Influence was possible; direct evidence that reporting was likely altered was absent.	Some concerns
Yentur; BASFI; 8 weeks	D5 Reported-result selection	The trial cites prospective registration (NCT06392620), but no dated detailed SAP was available and multiple outcomes, posture measures, within-group tests, and between-group analyses were eligible. Selection among analyses cannot be excluded.	Some concerns
Yentur; BASFI; 8 weeks	Overall	High risk in missing outcome data determines the overall judgment.	High

S6.3 ROBINS-I (original 2016 version) — non-randomized delivery-mode contrasts

Study/result	Domain	Key signaling answers and source-based rationale	Judgment
Fayet; total physical activity; remote vs hospital	D1 Confounding	Delivery mode was determined by calendar period: hospital delivery occurred in 2019–2020 and remote delivery in 2021 during the COVID-19 context. Participants were randomized to immediate versus waiting-list Tai Chi, not to mode. Age/sex matching cannot separate mode from pandemic, calendar, recruitment, and access effects; no valid adjustment can recover the delivery-mode contrast from non-overlapping eras.	Critical
Fayet; total physical activity; remote vs hospital	D2 Selection	Separate cohorts entered in different periods and participation in remote care may have depended on technology, preference, availability, or pandemic circumstances. Baseline characteristics were not fully presented by delivery mode.	Serious
Fayet; total physical activity; remote vs hospital	D3 Classification	Hospital and Zoom delivery were clearly defined and recorded prospectively; classification was not based on outcome information.	Low
Fayet; total physical activity; remote vs hospital	D4 Deviations	The intended Tai Chi protocol was similar, but adherence and retention differed materially; seven remote participants were reported lost. Deviations related to remote implementation were not adequately separated or adjusted.	Serious
Fayet; total physical activity; remote vs hospital	D5 Missing data	Flow and per-protocol denominators are difficult to reconcile by delivery cohort. Last-observation-carried-forward was used for ITT, without a robust model or sensitivity analysis for potentially informative remote attrition.	Serious

Study/result	Domain	Key signaling answers and source-based rationale	Judgment
Fayet; total physical activity; remote vs hospital	D6 Outcome measurement	Total physical activity was self-reported using ONAPS-PAQ by participants aware of mode and pandemic circumstances. Measurement method was the same, but recall/reporting could differ between calendar-defined cohorts.	Serious
Fayet; total physical activity; remote vs hospital	D7 Reported-result selection	Remote-versus-hospital delivery was exploratory rather than the randomized primary contrast. Multiple groups, time points, ITT/per-protocol analyses, and outcome summaries were available; no prespecified mode-effect estimand with a confidence interval was located.	Serious
Fayet; total physical activity; remote vs hospital	Overall	Critical confounding alone determines the overall judgment; the delivery effect cannot be separated from calendar period and COVID-19 context.	Critical
Bundle; BASFI; online yoga vs physiotherapy	D1 Confounding	Allocation depended on participant preference and whether physiotherapy was already being received. Motivation, digital access, disease activity, prior care, and other prognostic factors could differ; interventions also differed in content and minimum dose. Baseline-adjusted sensitivity analysis did not address all important measured or unmeasured confounders.	Serious
Bundle; BASFI; online yoga vs physiotherapy	D2 Selection	Selection into yoga or physiotherapy was linked to preference/existing treatment, factors plausibly related to adherence and outcomes. Follow-up analysis used participants with available measurements.	Serious
Bundle; BASFI; online yoga vs physiotherapy	D3 Classification	Physiotherapy was distinct, but the online yoga exposure combined live Zoom sessions and prerecorded videos; synchronous exposure was not separately classified. This materially limits a delivery-mode interpretation.	Serious
Bundle; BASFI; online yoga vs physiotherapy	D4 Deviations	Yoga attendance and home practice varied; physiotherapy content, intensity, and supervision were not standardized. Important co-interventions and deviations could differ, and the analysis did not isolate their effects.	Serious
Bundle; BASFI; online yoga vs physiotherapy	D5 Missing data	No imputation was used; mixed models were fitted to available outcome data and outcome-specific completeness varied. The report acknowledges incomplete data, but does not show evidence that missingness materially depended on true BASFI.	Moderate
Bundle; BASFI; online yoga vs physiotherapy	D6 Outcome measurement	BASFI was self-reported by unblinded participants; blinded outcome assessment was not implemented. Knowledge of self-selected intervention and different treatment intensity could influence reporting.	Serious
Bundle; BASFI; online yoga vs physiotherapy	D7 Reported-result selection	Many physical, activity-related, and psychological outcomes, alternative TSK definitions, adjusted/unadjusted models, and contrasts were available. Registration was reported, but no sufficiently detailed dated analysis plan was located to rule out result-dependent selection.	Serious
Bundle; BASFI; online yoga vs physiotherapy	Overall	At least one domain was serious and none was critical. Confounding, selection, mixed delivery classification, non-standardized comparator care, and unblinded self-report substantially limit causal interpretation.	Serious
